# Influence of Maternal Height and Weight on Low Birth Weight: A Cross-Sectional Study in Poor Communities of Northeastern Brazil 

**DOI:** 10.1371/journal.pone.0080159

**Published:** 2013-11-11

**Authors:** Revilane Parente de Alencar Britto, Telma Maria Toledo Florêncio, Ana Amelia Benedito Silva, Ricardo Sesso, Jairo Calado Cavalcante, Ana Lydia Sawaya

**Affiliations:** 1 Programa de Pós-graduação em Ciências Endocrinológicas, Escola Paulista de Medicina, Universidade Federal de São Paulo, Vila Clementino, São Paulo, São Paulo, Brazil; 2 Faculdade de Nutrição, Universidade Federal de Alagoas, Campus A. C. Simões, Maceió, Alagoas, Brazil; 3 Universidade de São Paulo, Escola de Artes, Ciências e Humanidades, Ermelino Matarazzo, São Paulo, São Paulo, Brazil; 4 Department of Medicine, Division of Nephrology, Escola Paulista de Medicina, Universidade Federal de São Paulo, Vila Clementino, São Paulo, São Paulo, Brazil; 5 Faculdade de Medicina, Universidade Federal de Alagoas, Campus A. C. Simões, Maceió, Alagoas, Brazil; Univ Mississippi Medical Center, United States of America

## Abstract

**Background:**

Low birth weight (LBW) is associated with an increased risk of mortality, adverse metabolic conditions, and long-term chronic morbidities. The relationship between LWB and short maternal stature coupled with nutritional status was investigated in poor communities.

**Methods/Principal Findings:**

A cross-sectional population-based study involving 2226 mother-child pairs was conducted during the period 2009-2010 in shantytowns of Maceió, Alagoas, Brazil. Associations between LBW and maternal sociodemographics, stature and nutritional status were investigated. The outcome variable was birth weight (< 2500g and ≥ 2500g). The independent variables were the age, income, educational background, stature and nutritional status (eutrophic, underweight, overweight and obese) of the mother. The frequency of LBW was 10%. Short-statured mothers (1^st^ quartile of stature ≤ 152cm) showed a tendency of increased risk of LBW children compared to mothers in the 4^th^ quartile of stature (>160.4cm) (OR: 1.42, 95% CI: 0.96 - 1.09, p = 0.078). Children from short-statured mothers weighed an average of 125g less than those from taller mothers (3.18±0.56kg *vs*. 3.30±0.58kg, respectively p = 0.002). Multivariate analyses showed that short stature, age < 20y (OR: 3.05, 95% CI:1.44 - 6.47) or were underweight (OR: 2.26, 95% CI:0.92 - 5.95) increased the risk of LBW, while overweight (OR: 0.38, 95% CI:0.16 - 0.95) and obesity (OR: 0.39, 95% CI:0.11 - 1.31) had lower risk for LBW. In taller mothers, lower income and underweight were associated with LBW (OR: 1.88, 95% CI: 1.07 - 3.29 and 2.85, 95% CI:1.09 - 7.47, respectively), and obese mothers showed a trend of increased risk of LBW (OR: 1.66, 95% CI:0.84 - 3.25).

**Conclusions/Significance:**

Overweight was found to have a protective effect in short-statured mothers, indicating that a surplus of energy may diminish the risk of LBW. Short-statured younger mothers, but not taller ones, showed higher risk of LBW. The mother being underweight, regardless of stature, was associated with LBW.

## Introduction

Globally, approximately 20 million infants are born with low birth weight (LBW < 2500 g) annually[1]. Approximately 95% of LBW infants are born in developing countries. The two main reasons for LBW are preterm birth (

< 37 weeks) and intrauterine growth restriction (IUGR). Low birth weight is considered to be one of the most significant factors associated with neonatal, post-natal and infant mortality as well as the increased risk of diseases in adulthood[2]. Determinants of LBW may differ between populations depending on whether LBW is due to growth restriction or prematurity. Sixty-four percent of the worldwide deaths of children under the age of five in 2010 were attributable to infectious causes, and preterm birth complications were the second-leading cause of death[3]. Preterm birth complications (14.1%), intrapartum-related complications (9.4%), and sepsis or meningitis (5.2%) were the leading causes of neonatal death. In older children, pneumonia (14.1%), diarrhea (9.9%), and malaria (7.4%) claimed the most lives[3]. Brazil is among the 10 countries with the highest number of preterm births[4]. 

One of the most important determinants of fetal growth and birth weight is maternal nutritional status[5-7]. Studies have found that short maternal stature increases the risk of LBW[8] and offspring mortality, underweight and stunting[9]. A recent systematic review and meta-analyses[10] found that short-statured women have a higher unadjusted risk of preterm birth and LBW, and tall women have approximately one half of the unadjusted risk of LBW compared to women of the reference height.

In addition, body mass index [BMI = weight (kg)/height (m)^2^] plays a critical role in the determination of gestational outcome, as maternal overweight and underweight are both strong predictors of birth weight[11,12]. A meta-analysis of 78 studies involving more than one million women in developed and developing countries revealed that maternal undernutrition was strongly associated with a greater risk of LBW[13]. On the other hand, a meta-analysis of 84 studies consisting of a similar number of women indicated that maternal overweight or obesity might reduce the risk of LBW and that the protective influence appeared to be stronger in developing countries[14], although the authors identified this association as spurious and due to bias in the analysis. Based on the above research, it is evident that maternal stature and BMI both contribute significantly to the birth weight of children, but no information is available regarding the impact of the combination of short stature and BMI on the risk of LBW.

The Brazilian state of Alagoas has the lowest human development index (0.677) in the country[15]. The population of Alagoas is estimated to be around three million[16], and 56.6% of the population live in absolute poverty; 32.2% live in extreme poverty[17]. Of the irregular settlements in the state (e.g., shantytowns and illegally occupied urban areas), 94.6% are located in the capital city of Maceió[18]. Previous studies conducted by our research group in the shantytowns of Maceió have revealed a high prevalence of individuals presenting with a combination of short stature and obesity, particularly women[19,20]. 

The aim of the present study was to examine the hypothesis that obese mothers of short stature are at increased risk of bearing children with LBW, as has been described previously for undernourished mothers of short stature. For this purpose, the association between LBW and short maternal stature, coupled with maternal current nutritional status, has been investigated in a sample population drawn from poor communities in Maceió-AL, Brazil. 

## Methods

The study was approved by the Ethics Committees in Research of the Federal University of Alagoas (Maceió, AL, Brazil; protocol number 20090132001-6) and of the Federal University of São Paulo (São Paulo, SP, Brazil; protocol number 1731/11). The aims and objectives of the study were explained to all potential participants, and it was emphasized that participation was voluntary. Written informed consent was obtained from the mothers for themselves and on behalf of the children participants prior to the commencement of the study. None of the invited participants refused to participate in the study.

### Study Population

The cross-sectional, population-based study was conducted during a one-year period (2009-2010) in shantytowns located on the periphery of Maceió. The necessary sample size was estimated using the StatCalc module in Epi Info™ (version 3.5.1.), assuming a LBW prevalence of 8%, a study power of 80% and a type one error of 5%. The minimum sample population required to comply with these criteria was estimated at 2972 children, i.e., 129 children per visited shantytown.

Data were collected in the homes of participants using a pre-tested structured questionnaire. In each shantytown, visits commenced at a previously selected corner starting point and continued in a clockwise direction. At each home, residents were asked if there was at least one child under 5 years of age living in the home. If there was no such child in the home, the researcher moved to the next dwelling until a sufficient number of households were sampled. Children with unconfirmed birth weight; children with genetic, endocrine or anatomical abnormalities that could compromise evaluation; pregnant or lactating women; mothers less than 18 or more than 49 years old; and non-biological mothers were excluded from the analysis. 

### Determination of Nutritional Status

Maternal nutritional status was based on BMI using the cutoff points recommended by the World Health Organization (WHO) as follows: underweight, BMI < 18.5 kg/m^2^; eutrophic, BMI 18.5 to <25 kg/m^2^; overweight, BMI 25 to < 30 kg/m^2^; and obese, BMI ≥ 30 kg/m^2^ [20]. Anthropometric measurements were taken with the mothers barefoot and wearing light clothes. Body weight was determined using a portable digital scale with 180 kg capacity and 100 g sensitivity, and height was determined with the individual in an orthostatic position with the aid of a portable stadiometer consisting of a non-extendable 2 m measuring tape divided into 0.1 cm increments. Short stature was adopted as an indicator of undernutrition in early life. For the purpose of this study, mothers were classified according to stature quartiles based on the sample population, with those in the 1^st^ quartile (height < 152 cm, equivalent to < 3^rd^ percentile for 19-year-old women according to WHO[21]), considered to be of short stature, while those in the 4^th^ quartile (> 160.4 cm, or > 43^rd^ percentile of the WHO reference) were considered to be the reference group for analyzing the variables of interest. Previous studies from our research group[22,23] have used this method of height stratification as an adjustment for the generally low socioeconomic status of this population. As described above, the highest quartile mean in the study sample did not reach the 50^th^ percentile of the WHO reference. In addition, our previous studies found significant relationships between adults with short stature (1^st^ quartile), the comorbidities and the nutritional status of children. 

The nutritional status of children was assessed from the weight and height/length measurements, which were standardized according to technical recommendations[24]. Children were stratified with respect to the anthropometric indices height-for-age and weight-for-height Z-scores (HAZ and WHZ, respectively), according to gender and age, utilizing the reference distribution proposed by WHO[24]. The data were calculated using WHO Anthro software [25]. Children presenting HAZ < -2.0 were classified as short stature, whereas those with WHZ > +1.5 and > +2.0 were classified as overweight and obese, respectively. Birth weights were obtained from the medical records of the children, and a cutoff value of 2,500 g was adopted for the definition of LBW.

### Statistical Analyses

The associations between LBW and maternal sociodemographics, stature and nutritional status were investigated. The mean differences in birth weight values were distributed according to the maternal stature quartiles and evaluated using one-way analysis of variance (ANOVA) and the Tukey post-hoc tests.

The dependent variable was birth weight, which was dichotomized as < 2500 g and ≥ 2500 g. The independent (explanatory) variables were the mother’s age at birth (

< 20 and ≥ 20 years), family income (≤ ½ and > ½ Brazilian minimum salary), length of schooling (< 4 and ≥ 4 years), stature and nutritional status (eutrophic, underweight, overweight and obese), according to the above-mentioned cutoffs). 

The associations between LBW and the independent variables were tested. Univariate analyses were based on the Pearson x^2^-test and on the crude odds ratios with 95% confidence intervals (95% CI). In multivariate analyses using logistic regression, the criterion for inclusion of independent variables in the model was whether they were associated with the dependent variable in the univariate analyses at the p ≤ 0.20 level. Only those independent variables with p < 0.05 remained in the final regression models. Adjusted odds ratios (95% CI) were calculated. As we detected a modification of the effect of maternal BMI by stature in relation to LBW, the analyses were stratified by maternal stature. We used the first and the fourth stature quartiles in these analyses to maximize the chance of detecting an effect due to differences in stature and to obtain a clear separation between the groups of low and normal stature. 

The differences were considered statistically significant when the probability of alpha error was less than 5% (*P* < 0.05). 

Statistical analyses were performed using Epi Info™, version 3.5.1, and Statistica™, version 11. 

## Results

In the present study, 2226 mother-child pairs were included from 23 shantytowns; 51% of the children were girls (1413), and 49% were boys (1349). The population was very homogeneous with respect to socioeconomic and educational levels (Table 1). The average monthly family income was Brazilian reals (R$) 452 ± 322, equivalent to US$ 224 ± 160, at an exchange rate of US$ 2.02, in September 2012. The majority of families (70%) reported a monthly income of less than one minimum Brazilian salary (R$ 622 or US$ 308), while a considerable number (25.5%) reported an even lower household income (

< ½ minimum salary). Fifty percent of the mothers had attended school for less than 4 years. Forty-six percent of the children were overweight or obese, and 5% were underweight. The mean birth weight of the children was 3230 ± 575 g (Table 1). Short stature was observed in 25% of the women. Slightly under half (47.5%) of the short-statured mothers were either overweight or obese. Ten percent of the children surveyed had LBW. The frequencies of LBW were 11.6% in girls and 8.1% in boys (Pearson x^2^-test, P = 0.003, OR: 1.460 CI 95%:1.13 - 1.88).

**Table 1:** Socioeconomic and anthropometrical characteristics of mothers living in the shantytowns (n = 2226, Maceió, State of Alagoas, Brazil, 2009-2010).

**Parameters**	**Value**
**Mothers**	
Age, yr	27.3 (13 - 55)
Length of schooling, yr	2.9 ± 1.3
Low level of education (< 4 years)	910 (40.9)
Low family income (< ½ Brazilian minimum salary)	568 (25.5)
Stature, m	1.56 ± 0.06
BMI, kg/m^2^	25.1 ± 5.0
Eutrophic (BMI ≥ 18.5 and BMI ≤ 25.0 kg/m^2^)	1110 (50.1)
Underweight (BMI < 18.5 kg/m^2^)	114 (5.1)
Overweight (BMI > 25.0 and BMI < 30.0 kg/m^2^)	653 (29.3)
Obese (BMI ≥ 30 kg/m^2^)	349 (15.7)
**Children**	
Birth weight, g	3230±575

Values are: median (min-max), mean± SD or N (%)

BMI=body mass index

The analysis of the sociodemographic characteristics of the mothers associated with LBW is shown in Table 2. Younger mothers (less than 20 years of age) had more than twice the risk of having LBW children, and lower family income (≤ ½ Brazilian minimum salary) was also associated with an increased risk (34%) of LBW. Regarding maternal nutritional status, underweight mothers were almost 3 times more likely to deliver children with LBW (*P*<0.0001), while a 32% lower chance was observed for overweight mothers compared to mothers with a normal body mass index. 

**Table 2:** Characteristics of mothers according to Low Birth Weight (LBW) condition of their children and LBW odds ratios (n = 2226, Maceió, State of Alagoas, Brazil, 2009-2010).

**Parameter**	**Low Birth Weight**		**LBW odds ratio (95% CI)**	P Value
	**Yes, N (%)**	**No, N (%)**		
Age, yr				
< 20	36 (18.0)	164 (82.0)	2.22 (1.50-3.28)	<0.0001
≥ 20†	181 (9.0)	1828 (91.0)		
Family Income				
≤ 0.5 min salary	68 (12.0)	500 (88.0)	1.34 (0.99-1.81)	0.061
> 0.5 min salary†	153 (9.2)	1503 (90.8)		
Schooling length, yr				
< 4	97 (10.7)	813 (89.3)	1.09 (0.81-1.49)	0.560
≥ 4†	89 (9.8)	817 (90.2)		
Body Mass Index				
Eutrophic†	115 (10.4)	996 (89.6)	1	
Underweight	25 (21.9)	89 (78.1)	2.43 (1.48-3.91)	<0.0001
Overweight	50 (7.7)	602 (92.3)	0.72 (0.51-1.01)	0.060
Obese	32 (9.2)	317 (90.8)	0.87 (0.57-1.31)	0.585
Stature				
1^st^ quartile (≤ 152 cm)	66 (11.8)	491 (88.2)	1.42 (0.96-2.09)	
4^th^ quartile (> 160. 4 cm) †	49 ( 8.7)	517 (91.3)		0.078

† reference category

To maximize the possibility of detecting the influence of maternal stature in LBW, we contrasted the first (stature less or equal to 152 cm) and fourth (above 160.4 cm) height quartiles (Table 2). Mothers in the first quartile of stature had a tendency of increased risk (42%) of having LBW children compared with those in the 4^th^ stature quartile (p=0.078).

Considering the relation between child birth weight and maternal stature, children of mothers in the 1^st^ quartile of stature weighed an average of 125 g less than those of mothers in the 4^th^ quartile (p= 0.002) (Table 3). The mean birth weights of children of mothers in the 1^st^, 2^nd^ and 3^rd^ quartiles of stature were not significantly different.

**Table 3:** Birth Weight of children living in shantytowns of Maceió, State of Alagoas, Brazil, stratified according to maternal stature quartiles.

**Maternal Stature**	**N**	**Birth weight, kg**
1^st^ quartile (≤ 152 cm)	557	3.18±0.56
2^nd^ quartile (>152 cm and ≤156 cm)	552	3.20±0.55
3^rd^ quartile (>156 cm and ≤ 160.4 cm)	551	3.24±0.61
4^th^ quartile (>160.4 cm)	566	3.30±0.58*#

*p=0.002 compared with 1^st^ quartile; #p=0.03 compared with 2^nd^ quartile

(n = 2226, Maceió, State of Alagoas, Brazil, 2009-2010).

Because we detected a modification of the effect of maternal BMI by stature in relation to LBW children, we stratified the analysis of factors influencing birth weight by maternal stature. Table 4 and 5 show the results from the univariate and multivariate analysis of factors associated with low birth weight stratified by maternal stature. Tables 4 and 5 correspond to the 1st and 4^th^ quartiles of maternal stature, respectively. 

**Table 4:** Univariate and multivariate analysis of factors associated with low birth weight, stratified by maternal stature.

			**univariate analysis**		**multivariate analysis**	
**Parameter**	**Low Birth Weight**		**LBW odds ratio**	**p-value**	**LBW odds ratio**	**p-value**
	**Yes, N (%)**	**No, N (%)**	(**95% CI**)		**(95% CI)**	
Age, yr						
<20	11 (21.2)	41 (78.8)	3.36 (1.60-7.08)	0.0014	3.05 (1.44-6.47)	0.003
≥20†	38 (7.4)	476 (92.6)				
Family Income						
≤0.5 min. salary	11 (8.7)	115 (91.3)	1.01 (0.44-2.32)	0.978		
>0.5 min salary†	38 (8.6)	402 (90.8)				
Length of schooling, yr						
<4	24 (10.5)	205 (89.5)	1.42 (0.75-2.69)	0.285		
≥4†	18 (7.6)	218 (92.4)				
Maternal Body Mass Index						
Eutrophic†	32 (10.4)	276 (89.6)				
Underweight	8 (23.5)	26 (76.5)	3.68 (1.57-8.67)	0.003	2.26 (0.92-5.95)	0.075
Overweight	6 (4.0)	144 (96.0)	0.36 (0.15-0.87)	0.023	0.38 (0.16-0.95)	0.037
Obese	3 (4.1)	71 (95.9)	0.41 (0.12-1.36)	0.143	0.39 (0.11-1.31)	0.125

† reference category

Low maternal stature (1^st^ quartile). Maceió, State of Alagoas, Brazil, 2009-2010.

**Table 5:** Univariate and multivariate analysis of factors associated with low birth weight, stratified by maternal stature.

			**univariate analysis**		**multivariate analysis**	
**Parameter**	**Low Birth Weight**		**LBW odds ratio**	**p-value**	**LBW odds ratio**	**p-value**
	**Yes, N (%)**	**No, N (%)**	(**95% CI**)		**(95% CI)**	
Age, yr						
<20	8 (18.2)	36 (81.8)	1.82 (0.80-4.12)	0.151		
≥20†	55 (10.9)	450 (89.1)				
Family Income						
≤0.5 min. salary	27 (16.6)	136 (83.4)	1.81 (1.06-3.07)	0.028	1.88 (1.07-3.29)	0.027
>0.5 min salary†	39 (9.9)	355 (90.1)				
Length of schooling, yr						
<4	27 (11.2)	215 (88.8)	0.78 (0.44-1.37)	0.389		
≥4†	28 (13.9)	174 (86.1)				
Maternal Body Mass Index						
Normal weight†	29 (10.9)	236 (89.1)				
Underweight	7 (23.3)	23 (76.7)	2.41 (0.99-5.88)	0.052	2.85 (1.09-7.47)	0.032
Overweight	14 (8.7)	147 (91.3)	0.63 (0.34 1.17)	0.145	0.78 (0.38-1.61)	0.502
Obese	16 (15.8)	85 (84.2)	1.53 (0.83-2.81)	0.172	1.66 (0.84-3.25)	0.140

† reference category

Normal maternal stature (4^th^ quartile ). Maceió, State of Alagoas, Brazil, 2009-2010.

In mothers of short stature (1^st^ quartile), univariate analysis showed that younger age at childbirth and underweight were significantly associated with LBW (OR: 3.36 and 3.68, respectively), but overweight decreased the risk of giving birth to a child with LBW compared with normal BMI (OR: 0.36, 95% CI: 0.15-0.87) (Table 4). Multivariate analysis confirmed these results, as LBW remained associated with maternal age below 20 years (OR= 3.05, 95% CI: 1.44 - 6.47), although the effect of underweight was attenuated (OR: 2.26, 95% CI: 0.92-5.95). Overweight mothers had a significantly lower risk of LBW (OR: 0.38), and the same trend of decreased risk was observed in obese mothers, although this effect was not statistically significant. 

The results for the mothers in the 4th stature quartile are presented in Table 5. The univariate analysis revealed that lower family income and being underweight were related to LBW (OR: 1.81 and 2.41, respectively), and obese, but not overweight, mothers showed a tendency towards increased risk of LBW. Age, family income, BMI, underweight, overweight and obesity were then included in the multivariate analysis. The results confirmed a significant association between both lower family income and underweight and LBW (OR: 1.88 and 2.85, respectively). The data from obese mothers demonstrated a similar trend, although not statistically significant, for increased risk of LBW (OR: 1.66, 95% CI: 0.84-3.25).

## Discussion

The causes of LBW are different in developing and developed countries[1]. In developed countries, preterm birth is major cause of LBW, whereas in developing nations, most LBW newborns are born at term but are small for gestational age (SGA)[26]. Because it is relatively difficult to obtain accurate gestational age estimates in very low income communities, we used birth weight to compare the nutritional status of the mother to the birth conditions of the child. The prevalence of LBW (10%) in this study population from shantytowns around the state capital Maceió was 17.5% higher than that reported for the state of Alagoas (7.6%) and 15% higher than the average in Brazil (8.4%)[27]. The prevalence of LBW in Brazil has remained stable (approximately 8%) since 2000 despite an increase in the number of preterm births. One possible explanation for this stability is the reduction in the frequency of intrauterine growth retardation, which counterbalances the negative effects of increased premature births[28]. The prevalence of LBW within Brazil is, however, somewhat perplexing in that the richer and more developed areas exhibit a higher prevalence of LBW than the less privileged regions. It has been suggested that this apparent paradox may result from inadequate recordkeeping and the lower survival rate of premature babies in the poorest municipalities. In isolated areas of the country, data such as gestational age, birth weight, number of home births and number of live births may be recorded incorrectly or not at all. Indeed, it is estimated that the frequency of underreporting of birth events in Brazil is approximately 7.2%[29,30]. 

Various studies have shown that poor families in different parts of the world have a greater risk for children with LBW than their more affluent counterparts[31-34]. A recent report from the United States confirmed that low income and income inequality exert a negative effect on birth weight[35]. The present study corroborates these findings because family income was found to be inversely associated with LBW throughout the entire sample and for normal stature mothers in the 4^th^ quartile (OR: 1.34, 95% CI: 0.99-1.81, P = 0.06 and adjusted OR: 1.88, 95% IC: 1.07-3.29, P = 0.027, respectively), although other factors were more relevant as determinants of LBW in short-statured mothers. In addition to being an extremely poor population, the average height of all mothers was low, 156 cm, which corresponds to the 15th percentile of the WHO reference for 19-year-old girls [21]. For this reason, we divided the population into quartiles of stature and compared the lowest quartile to the highest quartile. A significant negative effect on the birth weight of children was observed when comparing mothers in the first and second quartiles of stature to those in the fourth quartile of stature.

No association between length of schooling and LBW could be established in the present study, most likely because the sample population was very homogeneous with respect to this variable. The mean length of schooling was 2.9 years, and more than 40% of the mothers had less than 4 years of education. However, the association between poor maternal education and LBW has been observed in developed and developing countries and is well described in the literature[8,36]. A study of 78,582 children from Rio de Janeiro, Brazil, concluded that both level of education and maternal age were strongly associated with LBW[37]. 

Along with the factors mentioned above, gender can also influence the fetal weight[38,39]. The results presented herein revealed that the risk of LBW was 46% higher in girls, a finding that agrees with those from previous studies conducted in the southern Brazilian state of Paraná[40] and in Indonesia[41].

The results of the present study confirmed that young mothers (

< 20 years old) were 2.2 times more likely to give birth to children with LBW than their older counterparts when the entire sample was considered. This association became insignificant when mothers in the fourth quartile of stature were assessed, while younger short-statured mothers were 3.1 times more likely to give birth to LBW infants. This finding demonstrates a stronger likelihood of giving birth at an early age among short-statured mothers.

Maternal BMI is known to be a determining factor for fetal development in that both excess weight and weight deficiency can lead to adverse gestational outcomes[12,13,42]. A cohort study demonstrated that maternal underweight not only increased the risk of LBW but also the risk of premature births and neonatal mortality[43]. Additionally, a study of more than 2 million children and 751,912 low- and medium-income mothers from 54 emerging countries[9] found a direct relationship between short maternal stature and increased LBW, perinatal mortality and stature deficit in their children. 

The present study confirmed a strong association between LBW and maternal underweight, regardless of stature, as underweight mothers were 2.43-fold more likely to have LBW offspring than the group as a whole. On the other hand, a marginal protective effect of overweight in mothers was found (OR; 0.72, 95% CI: 0.51-1.01, P = 0.060). As the effect of BMI appeared to be different in mothers of short and normal stature, we carried out an analysis stratified according to the 1^st^ (short) and 4^th^ (normal) stature quartiles. In short-statured mothers, age and underweight were associated with LBW; overweight mothers were protected (adjusted OR: 0.38, 95% CI: 0.16-0.95, P = 0.037) against having LBW children, and this tendency was also observed among obese mothers. Among mothers in the 4^th^ stature quartile, income and underweight were the factors associated with LBW in the multivariate analysis; however, in this subgroup, being overweight or obese was not significantly associated with protection against LBW. Obese mothers showed a trend towards increased risk of LBW (OR: 1.66, 95% CI: 0.84-3.25, P = 0.14). These results show that, in addition to the association between LBW, maternal short stature and undernutrition that has been described in the literature, mothers who were overweight at the time of the study were less likely to have babies with LBW. We can speculate that in extreme poverty, overweight acts as a protective factor and ensures better health for the children of mothers with short stature. This finding is the opposite of those described for developed countries such as England and the United States, where overweight (BMI > 25 kg/m^2^) non-short mothers were observed to have a higher risk of adverse fetal and perinatal complications and infant death [44,45]. Among mothers with normal stature, the results of the present study, although not significant, indicated a higher risk of LBW in those with obesity at the time of the study. This finding is in line with the literature results concerning the harmful consequences of maternal obesity on fetuses and infants in developed countries[44,45] .

It is well known that adult short stature reflects health processes throughout life and is associated with nutritional stress during the early stages of life[8,9,46]. The biomechanical characteristics of mothers of short-stature must also be considered because this condition is associated with a narrower pelvis, which increases the likelihood of cephalopelvic disproportion and obstructed labor[9]. Moreover, a short-statured mother may not be able to supply sufficient amounts of nutrients to the fetus, a situation that results in greater susceptibility to a negative gestational outcome. Within this context, overweight short-statured mothers have a surplus of energy and/or nutrients that may protect the fetuses. 

The present study reports the effects of combined maternal short stature and overweight or obesity on the birth weight of children for the first time . The coexistence of stature deficit and excess weight is being observed at an increasingly early age in developing countries. A study conducted in 2001 established that the prevalence of overweight and obese women of short stature in poor communities of Alagoas was 30%[20], while a more recent investigation revealed that the phenomenon of stunting in combination with obesity had become prevalent in underprivileged children from the same area[47]. In the present study, we observed that the frequency of overweight and obesity was 20% higher when combined with short stature (47.6%) than when combined with normal stature (39.5%).

Our study has several limitations: the lack of an assessment of other risk factors for low birth weight, such as smoking status and alcohol intake, parity, and other maternal and obstetric conditions; the inability to discriminate between at-term and premature children; and the cross-sectional study design that prevented us from establishing a clearer causal relationship between the mothers’ BMI and LBW. Mothers’ BMI was determined an average of 30 months following childbirth. We believe that it is unlikely that maternal BMI changed dramatically after childbirth, as the establishment of obesity, overweight or even undernutrition in adult, low-income women usually requires several years and is directly related to their long-term living conditions. 

In conclusion, the most important finding of this study was that overweight short-statured mothers showed a significantly lower risk of LBW. Thus, overweight seemed to be a protective factor against LBW in extreme poverty conditions. In addition, younger mothers had a higher risk of LBW and the effect was stronger among those with short stature, as the effect was not significant among normal-stature mothers when analyzed alone. Income had a negative impact on birth weight only in the normal-statured mothers, while in short-statured mothers, the negative effects of low age and the protective effects of overweight remained the most important factors in the multivariate analysis. Our other results are agree with the literature, as maternal underweight, regardless of stature, was associated with LBW, and taller women (4^th^ quartile) gave birth to significantly heavier babies compared those in the first and second quartile. Because this is a cross-sectional study, prospective studies are necessary to confirm the present findings. 
